# Twelve Lectures on the Structure of the Central Nervous System

**Published:** 1891-03

**Authors:** 


### Book Reviews.

---

TWELVE LECTURES ON THE STRUCTURE OF THE CENTRAL NERVOUS SYSTEM, for physicians and students. By Dr. Ludwig Edinger, Frankfort on the Main. Second revised edition, with 133 illustrations. Translated by Willis Hall Vitturn, M. D., St. Paul, Minn. Edited by C. Eugene Riggs, M. D., Professor of Mental and Nervous Diseases, University of Minn., etc. Published by F. A. Davis, Phila., 1890.

Since the first works on anatomy, up to the present day, no work has appeared on the subject of the general and minute anatomy of the central nervous system so complete and exhaustive as this work of Dr. Ludwig Edinger. Being himself an original worker, and having the benefits of such masters as Stilling, Weigeit, Geilach, Meynert and others, he has succeeded in transforming the mazy wilderness of nerve fibres and cells into a district of well marked pathways and centres, and by so doing, has made a pleasure out of an anatomical bugbear.

As to the merits of the American edition in English, translated by Dr. Willis Hall Vitturn, we can say, that the translator has done his work well, for on comparing the work with the original, one can but acknowledge that so far as is compatible with a good translation, the work is verbatim.

C. Eugene Riggs, A. M., E. D. has shown much skill as an editor, in giving to us in English, the original in modo et forma. H. H.

